# Localized Osteomyelitis of the Mandible Secondary to Dental Treatment: Report of a Case

**DOI:** 10.5681/joddd.2009.016

**Published:** 2009-06-05

**Authors:** Saeed Nezafati, Mohammad Ali Ghavimi, Amir Saeed Yavari

**Affiliations:** ^1^Assistant Professor, Department of Oral and Maxillofacial Surgery, Faculty of Dentistry, Tabriz University of Medical Sciences, Tabriz, Iran; ^2^Post-graduate student, Department of Oral and Maxillofacial Surgery, Faculty of Dentistry, Tabriz University of Medical Sciences, Tabriz, Iran

**Keywords:** Arsenic, localized osteomyelitis, osteomyelitis of mandible, pulp devitalizer

## Abstract

Osteomyelitis of the jaws following dental treatment is a rare condition which usually occurs in immuno-compromised patients both locally and generally. A case is presented with the alveolar bone necrosis resulting from leakage of an arsenical devitalizing paste into the periodontium. The treatment procedures and the outcomes are discussed in this article.

## Introduction

Osteomyelitis of the jaws is now defined by the presence of exposed bone in the mouth, which fails to heal after appropriate intervention.^1^ Osteomyelitis is an inflammation of bone cortex and marrow that develops in the jaw usually after a chronic infection.^2-4^ The incidence of osteomyelitis has dramatically decreased since the introduction of antibiotics.^5^ Moreover, osteomyelitis of the head and neck skeleton is rare, particularly in the jaws.^3,4^

The medications linked to osteomyelitis are steroids, chemotherapeutic agents, bisphosphonates and other toxic therapeutic agents.^1,6-10^ Local conditions that adversely affect the blood supply or lead to tissue necrosis can also predispose the host to a bone infection or localized osteomyelitis.^10,11^

Osteomyelitis is diagnosed on the basis of patient history, clinical examinations, and the surgical and radiographic findings. Histopathologic examinations can be consistent with the diagnosis and the microbiologic tests can be helpful.^6,7^

Osteomyelitis has a range of clinical manifestations depending on the virulence of the infecting organisms, host resistance, and the reaction of the periosteum to inflammation.^12^ Osteomyelitis of the mandible following routine dental treatment has rarely been reported in the literature.^10,11^ This article reports a case of a healthy patient who developed osteomyelitis of the lower jaw following root canal therapy.

## Case Report

A 24-year-old male patient without any systemic disorders was referred to the Department of Oral and Maxillofacial Surgery, Tabriz University of Medical Sciences, Tabriz, Iran, in December 2008 because of the left mandibular pain and swelling. The patient had no relevant history except for smoking for 5 years. The left mandibular first molar had been extracted 5 weeks before the examination. Three days before extraction, the tooth had undergone pulpotomy using an arsenic-based pulp devitalizer. This material is commonly used to accelerate pulpal necrosis and consequent pain relief by some non-academic practitioners. Four days after extraction the patient had experienced severe pain in the left mandibular region, which had progressively increased.

**Figure 1:**
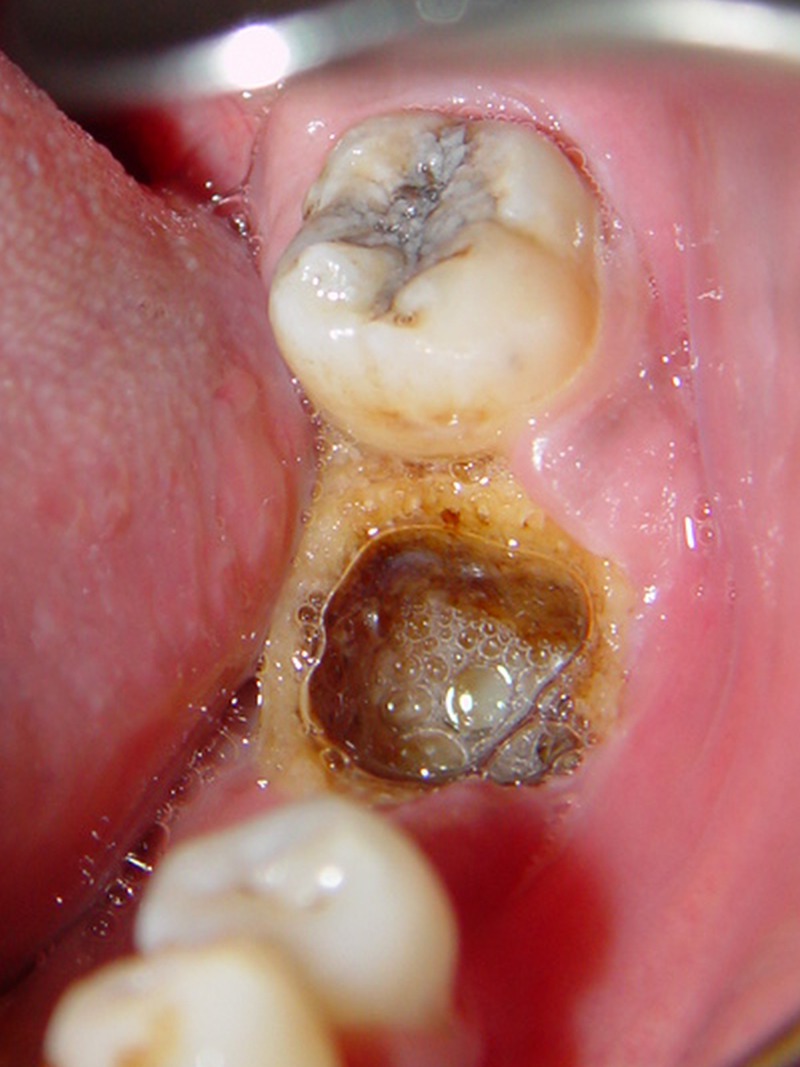
Clinical view of the exposed bone after tooth extraction.


**Figure 3:**
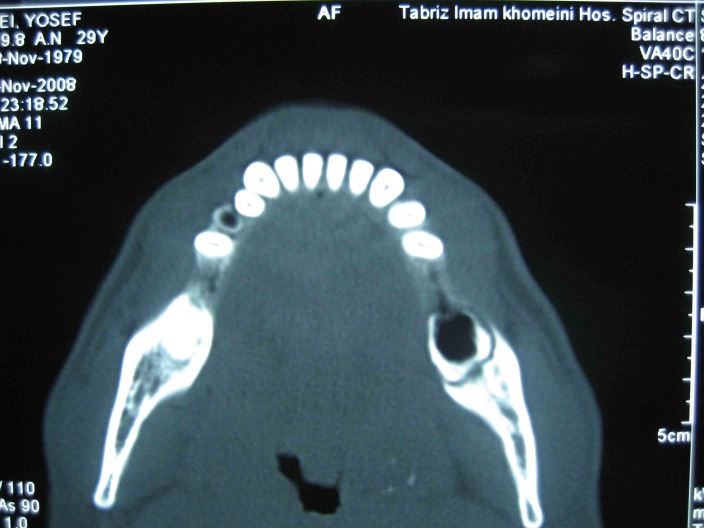
Preoperative axial CT-scan from extraction socket and surrounding bone.

Clinical examinations revealed spontaneous mandibular pain, tenderness, and a poorly healed socket with alveolar bone exposure (Figure 1). No fistula was detected in the adjacent mucosa or skin. The extraction socket and surrounding bone had a moth-eaten appearance and there was evidence of sequestrum formation on conventional x-rays and CT scans (Figures 2 and 3).

Figure 2. The preoperative radiographic view of extraction socket (a). The necrotic bone had been surrounded by a radiolucent band (arrow) (b).

**a:**
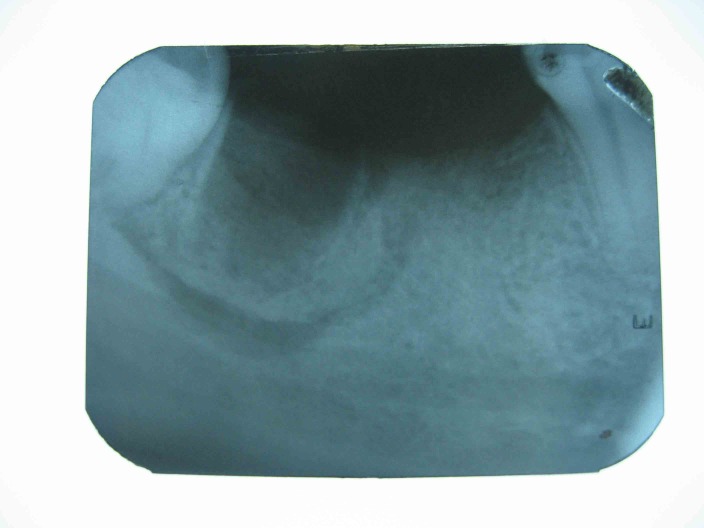

**b:**
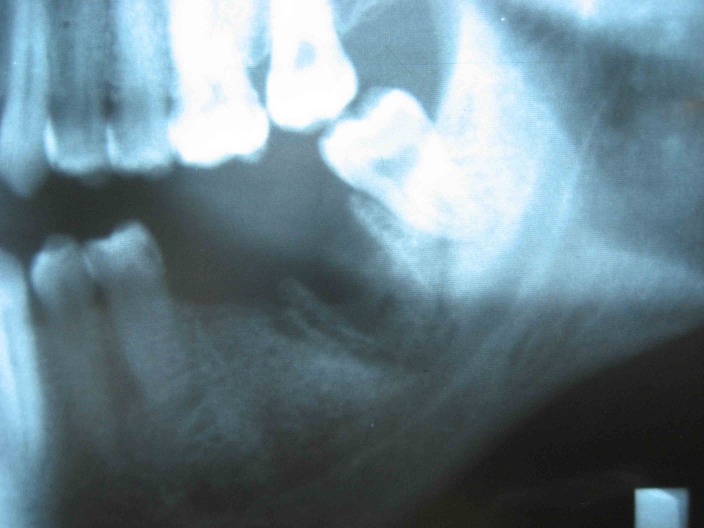

The hematologic profile showed an increase in white blood cell count; however, the red blood cell count was normal. The Erythrocyte Sedimentation Rate (ESR) had risen (40 mm first hour).

With the clinical diagnosis of osteomyelitis, the patient was given intravenous penicillin 3,000,000 units every 3 hours and was scheduled to the operating room for sequestrectomy and exploration. The exposed bone was removed and complementary curettage and irrigation was performed. The necrotic bone was sent for histopathologic examination. The postoperative period was uneventful and the patient was discharged from the hospital with oral penicillin 500 mg 4 times a day for the next two weeks. The histopathologic view showed necrotic bone with acute inflammatory cells (Figures 4). The patient was symptom-free in the first postoperative follow-up one month after the surgery. The patient refused to undergo postoperative control x-rays.


Figure 4. (a) Histopathologic view of bone segment with fibrovascular tissue and infiltration of acute inflammatory cells (×10). (b) Necrotic bone with acute inflammatory cells (×40).

**a:**
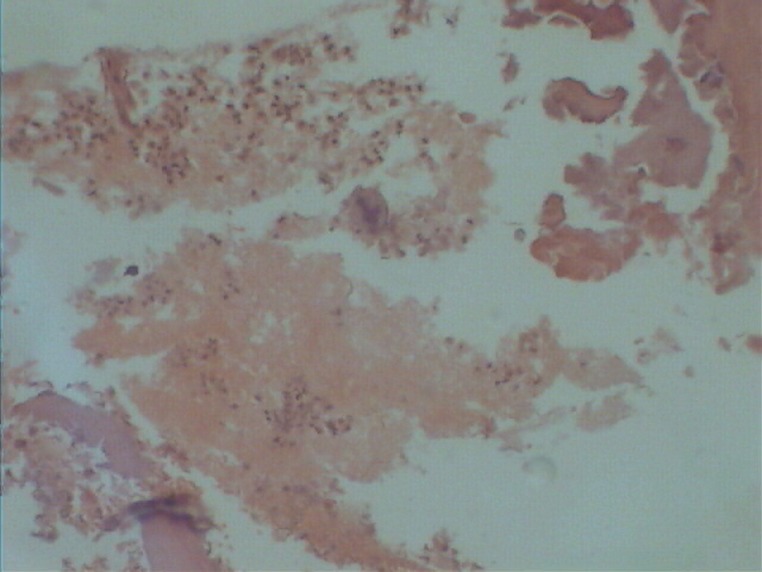

**b:**
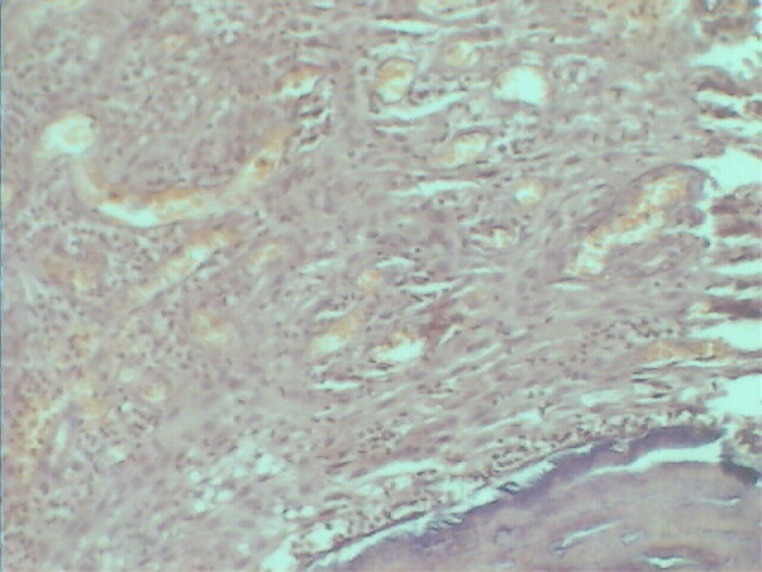

## Discussion

Osteomyelitis of the jaws is a rare condition, which has been associated with multiple systemic diseases including diabetes, autoimmune states, malignancies, malnutrition, and acquired immunodeficiency syndrome. The medications linked to osteomyelitis are steroids, chemotherapeutic agents, and bisphosphonates.^3^

There are many therapeutic materials affecting bone metabolism.^7,9^ Some of them are used traditionally in old dentistry. One of the examples is arsenic-based compounds. These materials are popular among practitioners who are not familiar with new anesthesia techniques of contemporary dentistry. In the past, local anesthesia was less reliable and the use of devitalizing pastes was an established practice. Arsenic reduces pulp sensitivity by necrotizing the nerve endings of dental pulp. However, arsenic and its compounds are extremely toxic and potentially carcinogenic when placed in contact with the hard and soft tissues of the body. Caution should be exercised during cavity preparation not to perforate the floor of pulp chamber, which could provide an easy and direct route for leakage of the material. To avoid the risk of arsenical necrosis, the manufacturer strongly recommends that no pressure should be exerted on the pellet or during sealing the cavity hermetically with temporary cement.

The osteomyelitis seen in this case occurred after condensation of arsenic trioxide into the pulp chamber and the canals of the tooth. It is probable that the material passed beyond the apex of the tooth following heavy condensation or probably the material penetrated into the peridental tissues because of loose or inappropriate temporary filling. Treatment of osteomyelitis of the jaws includes elimination of the cause, incision and drainage, sequestrectomy, saucerization, decortication, resection of the jaw, antibiotics and hyperbaric oxygen.^5^

The main treatment of localized osteomyelitis in a patient without any systemic conditions is to remove the etiology of the disease as well as antibiotic therapy to prevent post-surgical infection.^3^ Antibiotic therapy should be instituted at the earliest moment and can be changed according to the results of antibiogram.^13^ In the present case treatment plan included removal of the localized necrotic bone and sequestrum. Antibiotic therapy was instituted prior to the surgery with intravenous penicillin and continued postoperatively to prevent post-surgical infection. Post-treatment evaluations showed complete healing.

## Conclusion

Arsenic compounds have no place in contemporary dental practice and dentists should be warned against their potential hazards and adverse effects.

## References

[1] Reid IR (2009). Osteonecrosis of the jaw: who gets it, and why. Bone.

[2] Yeoh SC, Macmahon S, Schifter M (2005). Chronic suppurative osteomyelitis of the mandible: case report. Aust Dent J.

[3] Kushner G M, Alpert B (2003). Osteomyelitis and osteoradionecrosis. In: Miloro M, Ghali GE, Larsen PE, Waite PD, eds. Peterson's Principles of Oral and Maxillofacial Surgery.

[4] Fonseca RJ, Turvey TA, Betts NJ (2000). Oral and Maxillofacial Surgery.

[5] Barry CP, Ryan CD, Stassen LF (2007). Osteomyelitis of the maxilla secondary to osteopetrosis: a report of 2 cases in sisters. J Oral Maxillofac Surg.

[6] Senel FC, Saracoglu TEKIN U, Durmus A, Bagis B (2007). Severe osteomyelitis of the mandible associated with the use of non-nitrogen-containing bisphosphonate (disodium clodronate): report of a case. J Oral Maxillofac Surg.

[7] Dimitrakopoulos I, Magopoulos C, Karakasis D (2006). Bisphosphonate-induced avascular osteonecrosis of the jaws: a clinical report of 11 cases. Int J Oral Maxillofac Surg.

[8] Dimitrakopoulos I, Magopoulos C, Katopodi T (2007). Mandibular osteomyelitis in a patient with pyknodysostosis: a case report of a 50-year misdiagnosis. J Oral Maxillofac Surg.

[9] Hino S, Murase R, Terakado N, Shintani S, Hamakawa H (2005). Response of diffuse sclerosing osteomyelitis of the mandible to alendronate: follow-up study by 99mtc scintigraphy. Int J Oral Maxillofac Surg.

[10] Yavuz MS, Kaya GS, Yalçin E, Aras MH (2008). Mandibular bone necrosis caused by use of arsenic paste during endodontic treatment: two case reports. Int Endod J.

[11] Ozmeriç N (2002). Localized alveolar bone necrosis following the use of an arsenical paste: a case report. Int Endod J.

[12] Jones J, Amess TR, Robinson PD (2005). Treatment of chronic sclerosing osteomyelitis of the mandible with calcitonin: a report of two cases. Br J Oral Maxillofac Surg.

[13] Clover MJ, Barnard JD, Thomas GJ, Brennan PA (2005). Osteomyelitis of the mandible during pregnancy. Br J Oral Maxillofac Surg.

